# Generative large language models in the clinical management of Alzheimer’s disease and mild cognitive impairment

**DOI:** 10.1007/s10072-026-09239-2

**Published:** 2026-07-13

**Authors:** Yosef Adiniaev, Mahmud Omar, Oved Daniel, Tohar M. Timor, Yiftach Barash, Olga R. Brook, Eyal Klang, Alon Gorenshtein

**Affiliations:** 1 Faculty of Medicine, University of Debrecen, Debrecen, Hungary; 2 BRIDGE GenAI Lab, Beth Israel Deaconess Medical Center, Harvard Medical School, 330 Brookline Ave, Boston, MA 02115 USA; 3 The Windreich Department of Artificial Intelligence and Human Health, Mount Sinai Medical Center, New York, NY USA; 4 The Hasso Plattner Institute for Digital Health at Mount Sinai, Mount Sinai Health System, New York, NY USA; 5 Neurology Division, Tel Aviv Sourasky University Medical Center, Tel Aviv, Israel; 6 Department of Radiology, Beth Israel Deaconess Medical Center, Harvard Medical School, Boston, MA USA; 7 Department of Neurology, Beth Israel Deaconess Medical Center, Harvard Medical School, Boston, MA USA

**Keywords:** Large language models, Alzheimer's disease, Mild cognitive impairment, Systematic review, Clinical decision support, Patient education

## Abstract

**Background:**

Dementia affects over 55 million people worldwide. Mild cognitive impairment (MCI) often precedes Alzheimer’s disease (AD). Clinical management requires integrating uncertain evidence from neuropsychological testing, neuroimaging, and biomarkers. Large language models (LLMs) also generate probabilistic outputs, but whether they can reliably support diagnostic, therapeutic, or educational tasks in AD and MCI has not been systematically examined.

**Methods:**

We searched PubMed, Scopus, and PubMed Central (January 2023 to April 2026) for studies evaluating generative LLMs on clinical tasks in Alzheimer’s disease (AD) or mild cognitive impairment (MCI). Risk of bias was assessed using QUADAS-AI and AXIS. Narrative synthesis followed the SWiM guideline. PROSPERO: CRD420261372436.

**Results:**

Eleven studies were included: diagnosis (*n* = 3), treatment guidance (*n* = 2), and patient/caregiver education (*n* = 8); two studies contributed to multiple domains. Diagnostic models achieved high internal accuracy (0.94–0.97) but declined on external validation; three-way classification accuracy dropped approximately 7% points, and MMSE-prediction R² collapsed from 0.90 to 0.25 on an external dataset. Treatment guidance approached but did not match structured clinical guidelines. Educational outputs were rated moderate to high quality but lacked source attribution and exceeded recommended reading levels; retrieval augmentation improved usability without improving accuracy. Hallucination was quantified in only 2 of 11 studies, and no study evaluated prospective clinical use.

**Conclusions:**

Current evidence does not support the use of LLMs for diagnosis, treatment selection, or patient education in AD/MCI without clinician oversight. These findings reflect the specific model versions, prompting strategies, and evaluation conditions in place at the time of each study, and are further limited by small heterogeneous evaluations, sparse hallucination measurement, and absence of prospective clinical validation.

**Supplementary Information:**

The online version contains supplementary material available at 10.1007/s10072-026-09239-2.

## Introduction

Alzheimer’s disease (AD) is the leading cause of dementia, which affects over 55 million people worldwide [1]. Mild cognitive impairment (MCI) often precedes AD [2, 3].

There is no definitive in vivo test for AD; clinicians integrate converging evidence from neuropsychological testing, structural and functional neuroimaging, CSF biomarkers, and amyloid PET [4–6]. Management involves similar uncertainty: treatment is calibrated to clinical staging that is itself uncertain, caregiver education must be tailored to a trajectory that varies between individuals, and guidelines are revised as new evidence shifts thresholds for intervention [4, 7, 8]. The interpretive, communicative, and educational work this entails places a substantial burden on clinicians and caregivers alike [9].

Large language models (LLMs) are generative AI systems that produce text by predicting token sequences from patterns learned across large corpora [10]. They have been applied to medical question-answering, clinical note summarisation, and patient communication across a range of specialties [11, 12]. LLMs can also be paired with vision encoders to incorporate imaging-derived information [10]. However, LLMs may produce fluent but factually incorrect outputs (hallucinations), and this failure mode is most consequential when the clinical task requires a single correct answer [11, 12]. In AD and MCI, the stakes are disease-specific: anti-amyloid therapies require biomarker-confirmed amyloid pathology and APOE ε4 genotyping for risk stratification; cholinesterase inhibitor prescribing demands cardiac comorbidity screening; and caregiver education must be calibrated to disease stage and trajectory [7, 8]. An LLM error in any of these contexts could alter eligibility for treatment, delay safety monitoring, or misinform patients about prognosis.

Earlier reviews of AI in dementia have focused on traditional machine learning and imaging classifiers [13] rather than on generative LLMs. Broader evaluations of LLMs in medicine address either single application domains or all of clinical medicine without disease specificity [14, 15]. Neurology-adjacent reviews have grouped AD with Parkinson’s disease and other conditions, diluting the signal. No systematic review has examined LLMs across the AD and MCI care continuum (diagnosis, treatment guidance, and patient and caregiver education) using current reporting standards.

We therefore conducted a systematic review to evaluate the performance and clinical relevance of LLM outputs across diagnostic, therapeutic, and educational tasks in AD and MCI, compare findings across models and study designs, and identify methodological gaps and safety concerns relevant to clinical implementation.

## Methods

### Protocol and registration

This review followed PRISMA 2020 guidelines [16]. The protocol was registered on PROSPERO (CRD420261372436) on 17 April 2026. Because PROSPERO registration occurred after title/abstract screening but before data extraction, the review was not prospectively registered.

### Data sources and searches

We searched PubMed, Scopus, and PubMed Central for studies published from 1 January 2023 through 18 April 2026. Search terms combined LLM-related and AD/MCI-related keywords (including free-text synonyms and MeSH terms where supported), limited to English-language records. Full search strings are provided in the Supplementary Materials. We conducted forward and backward citation searches.

### Study screening

We included studies that evaluated LLMs in the context of AD or MCI. We accepted studies that assessed LLMs for diagnosis, treatment recommendations, patient or caregiver education, guideline adherence, or clinical decision support.

Studies were required to report quantitative or expert-evaluated performance data. We included peer-reviewed English full-text articles only. We excluded studies focused on other dementia subtypes without an AD or MCI focus, as well as studies evaluating traditional machine learning, imaging-only AI, or rule-based NLP without an LLM component. Editorials, commentaries, letters without original data, conference abstracts, preprints, and study protocols were also not eligible. Three studies using generic “dementia” cohorts [17–19] were retained because their clinical tasks are directly applicable to AD and MCI care. Studies using fine-tuned models with an LLM core (e.g., LLaVA, FLAN-T5) were included even when the final output was a classification rather than generated text, because the underlying language model is what distinguishes these systems from conventional imaging classifiers.

Two reviewers (Y.A., A.G.) independently screened titles and abstracts using Rayyan [20]. Articles flagged for potential inclusion by either reviewer moved to full-text review. Disagreements were resolved through a third reviewer (E.K.).

### Data extraction

A standardised extraction form was piloted on three studies and applied to all included articles. One reviewer (Y.A.) extracted data and a second (A.G.) verified all entries; disagreements were resolved through discussion. We collected study identification, design, AD and MCI specificity, LLM model and version, customisation strategy, clinical task, comparator, outcome instruments, key results, and limitations. Studies contributing to more than one domain were extracted separately for each.

### Risk of bias assessment

Diagnostic-accuracy studies were assessed with QUADAS-AI [21], and non-diagnostic cross-sectional studies with AXIS [22]. Two reviewers (Y.A., A.G.) independently assessed each study; disagreements were resolved through discussion. Complete checklists are provided in the Supplementary Materials.

### Synthesis of findings

Meta-analysis was not feasible due to heterogeneity in LLM models, application tasks, outcome instruments, and reference standards. We performed a narrative synthesis following the SWiM reporting guideline [23], grouping studies by application domain and structural similarity within each domain. Studies addressing more than one domain contributed to each relevant grouping. Formal GRADE assessment was not applied. Given heterogeneity in outcome measures and evaluation frameworks, results were interpreted descriptively without cross-study standardisation.

## Results

### Study selection

**Fig. 1:**
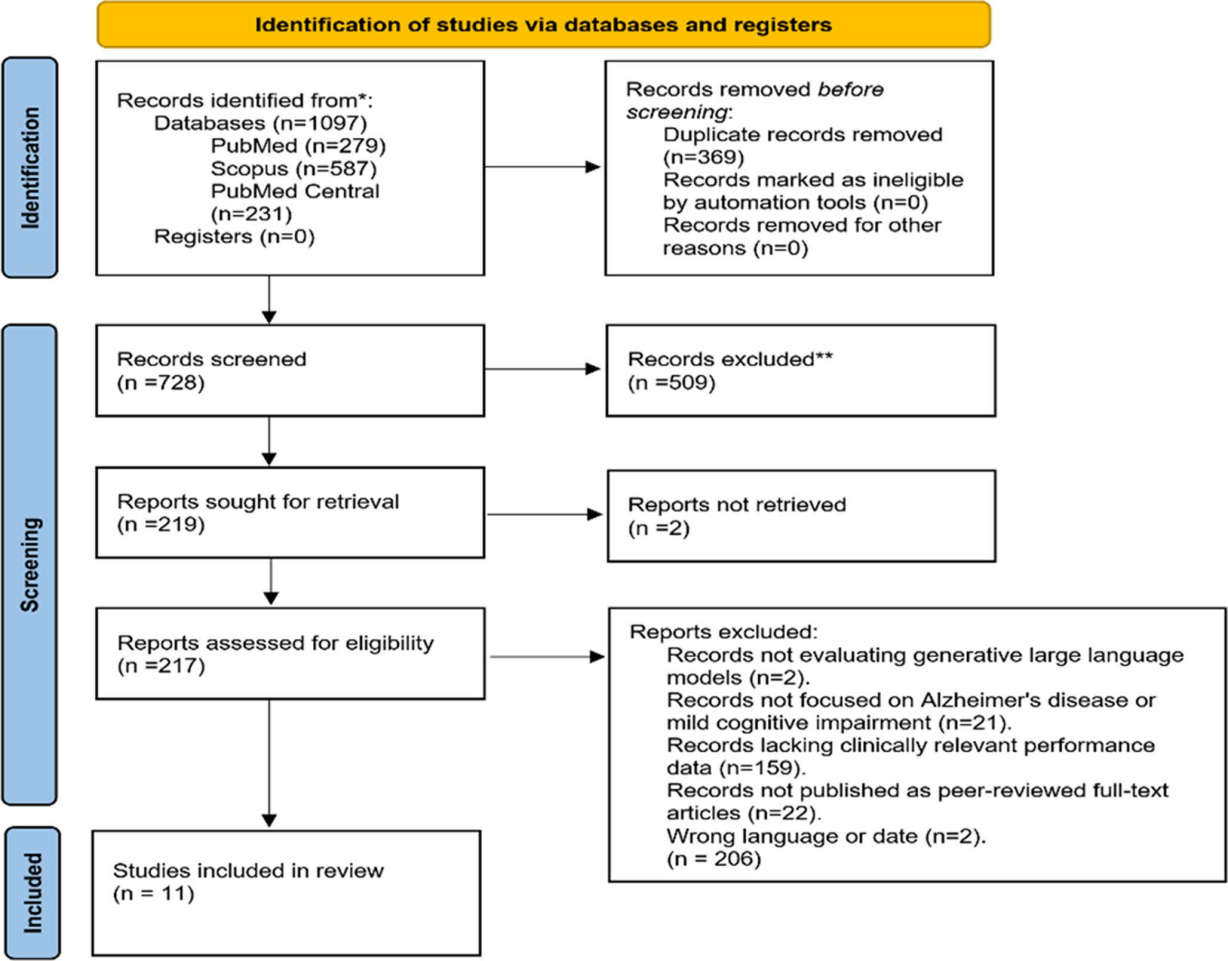
PRISMA 2020 flow diagram of the study selection process

The search identified 1,097 records across PubMed (279), Scopus (587), and PubMed Central (231). After removing 369 duplicates, 728 unique records were screened by title and abstract, of which 219 were sought for retrieval. Two reports could not be retrieved (full text unavailable). Of the 217 reports assessed for eligibility, 206 were excluded, most commonly for lacking clinically relevant performance data (*n* = 159), wrong publication type (*n* = 22), or populations outside AD and MCI (*n* = 21), other reasons (*n* = 4). Eleven studies were included in the final synthesis (Fig. 1). Forward and backward citation searches of the 11 included studies identified no additional eligible records.

### Study characteristics

The 11 included studies were published between 2024 and 2026 and originated from five countries. Ten used cross-sectional designs and one used a mixed-methods design. ChatGPT variants were evaluated in 9 of 11 studies; other models included LLaMA variants, FLAN-T5, Gemini, DeepSeek, Kimi, and ThinkAny (an AI search engine). Studies spanned three predefined application domains: diagnosis (Domain A, *n* = 3), treatment guidance (Domain B, *n* = 2), and patient and caregiver education (Domain C, *n* = 8); two studies contributed to multiple domains. Study characteristics, LLMs evaluated, and key findings are summarised in Table 1. The temporal and technical context of each evaluation, including model versions, testing dates, system type, external tool use, and guideline environment, is summarised in Supplementary Table S7.

**Table 1:** Characteristics and key findings of included studies, organised by application domain

First Author, Year	LLM(s)	Application	Key Finding	Sample
Domain A - Diagnosis (*n* = 3)				
Nie 2026	LLaVA-1.5 (fine-tuned)	MRI/PET classification (AD/MCI/CN)	ADNI 3-way acc 0.94; NACC 3-way 0.86 (7 pp drop); OASIS 3-way 0.89 (5 pp drop). AD-vs-CN acc: ADNI 0.99, NACC 0.91, OASIS 0.95.	ADNI 8244 (2244 test); OASIS 1680 (external test); NACC 840 (external test).
Chen 2025	FLAN-T5 + ADFormer	MRI classification + MMSE prediction	ADNI acc 0.97, AUC 0.98; OASIS zero-shot acc 0.81 (AD-vs-CN binary; no MCI class in OASIS). MMSE R² collapse 0.90→0.25 externally.	ADNI 8315 volumes; OASIS 373
Zhang 2024	GPT-4; LLaMA-2 70B	MMSE/CDR extraction from EHR notes	GPT-4 acc 83–90% vs. LLaMA-2 68–75%. GPT-4: 3 hallucinations; LLaMA-2: 27.	765 sampled; 742 after API errors; 722 assigned to reviewers; 710 in final analysis (52,948 patients in source EHR)
Domain B - Treatment Guidance (*n* = 2)				
Maiti 2025*	ChatGPT-3.5; ThinkAny (AI search engine)	Guideline concordance (EAN dementia)	LLMs Likert 4.0-4.3 vs. guideline 4.5 vs. Google 3.1–3.7. Friedman *p* < 0.002 (Q2, Q3); Q1 *p* = 0.051.	3 questions; 7 evaluators
Xiao 2025*	ChatGPT-4o; Gemini 2.0; Kimi	Treatment guidance (MCI)	ChatGPT-EN highest (acc 4.67/5); Kimi lowest (3.93). EN> Chinese on accuracy (*p* = 0.03).	18 Tx questions; 3 HP + 2 CP per language (10 evaluators)
Domain C - Patient and Caregiver Education (*n* = 8)				
Hasan 2024	ADQueryAid (GPT-4 + RAG + KG); ChatGPT-3.5	Caregiver chatbot usability	RAG system CUQ 83.8 vs. baseline 72.5. Significant on informativeness (*p* = 0.005).	20 ADRD caregivers
Aguirre 2024	ChatGPT-3.5	Caregiver Q&A quality	93% factual accuracy; 0/60 fabricated; 0/60 harmful. Synthesis 96%, comprehensiveness 63%.	60 Reddit posts; 3 clinician raters
Tukur Jido 2025	GPT-4; DeepSeek V3; Gemini Flash 2.5	Readability comparison	FRE: GPT-4 28.6 > Gemini 22.1 > DeepSeek 11.6. All exceed 6th-grade threshold.	9 outputs total (3 conditions × 3 models); AD subset: 1 output per model (*n* = 3).
Zhou 2026	ChatGPT-4o (baseline vs. prompt-engineered)	Caregiver education quality	Prompt engineering improved actionability, satisfaction, relevance (*p* < 0.05); not accuracy.	12 experts; 32 scenarios
Maiti 2025*	ChatGPT-3.5; ThinkAny	Care coordination education	Q1 Likert: guideline vs. ThinkAny vs. ChatGPT 3.5 vs. Google (*p* = 0.051).	1 education question; 7 evaluators
Dosso 2024	ChatGPT (GPT-3.5)	AD FAQ quality vs. organisations	QUEST: organisations 21 vs. ChatGPT 16. ChatGPT scored 0 on Attribution. FKGL 13.7 vs. 10.9.	18 FAQ items; 3 AD organisations
Huang 2024	ChatGPT (GPT-4)	AD myth correction	Mean Likert 1.1/+2 (agreement). No “strongly disagree” responses. 6/10 evaluators are co-authors.	16 myths; 10 geriatricians
Xiao 2025*	ChatGPT-4o; Gemini 2.0; Kimi	MCI education (symptoms, CP support, rehab)	Domain means 4.00-4.11/5. ChatGPT-EN consistently highest. EN> Chinese (*p* < 0.001). HP > CP.	54 education questions; 3 HP + 2 CP per language (10 evaluators)

* Maiti 2025 and Xiao 2025 contribute to multiple domains and appear in each relevant section

Abbreviations: *acc* accuracy, *AUC* area under the curve, *CN* cognitively normal, CP care partner, *CUQ* Chatbot Usability Questionnaire, *EAN* European Academy of Neurology, *EHR* electronic health record, *EN* English, *FKGL* Flesch-Kincaid Grade Level, *FRE* Flesch Reading Ease, *HP* healthcare professional, *KG* knowledge graph, *MCI* mild cognitive impairment, *MMSE* Mini-Mental State Examination, *NR* not reported, *pp* percentage points, *RAG* retrieval-augmented generation, *Tx* treatment

### Risk of bias

Risk-of-bias assessments using QUADAS-AI (diagnostic studies, *n* = 3) and AXIS (non-diagnostic studies, *n* = 8) are summarised in Figure S1 and reported in Supplementary Table S2. Key concerns include test-set leakage in Nie et al. [24] (best-performing checkpoint selected on the ADNI test set); reference-standard contamination in Zhang et al. [25] (human-corrected ChatGPT output used as ground truth); evaluator independence in Huang et al. [26] (6 of 10 raters are co-authors); and small item pools in Tukur Jido et al. [27] (*n* = 3 per condition) and Maiti et al. [18] (*n* = 3 total).

### Synthesis of findings

#### Diagnosis

Three studies evaluated LLMs for diagnostic tasks. Two fine-tuned vision-language models to classify AD, MCI, and cognitively normal subjects from neuroimaging; these function as classifiers rather than generative tools [24, 28]. One evaluated off-the-shelf LLMs for extracting cognitive-exam scores from clinical notes [25].

Both vision-language models achieved high internal accuracy on ADNI (Table 1) but declined on external datasets: Nie et al. reported a 7% point drop in three-way accuracy on NACC [24], and Chen et al. reported AD-versus-CN accuracy of 0.81 on OASIS zero-shot — a binary task with no MCI class, limiting comparability with ADNI three-way results [28]. MMSE-prediction R² collapsed from 0.90 internally to 0.25 externally, indicating minimal predictive value outside the training distribution [28].

For information extraction, GPT-4 outperformed LLaMA-2 70B on both MMSE and CDR extraction (Table 1), producing 3 hallucinations versus 27 [25]. This was one of only two studies quantifying hallucination. The reference standard (human-corrected GPT-4 output) introduces bias favouring GPT-4, as the authors acknowledge [25].

#### Treatment guidance

Two studies evaluated LLMs for treatment-related guidance [18, 29]. Both found that LLMs outperformed search engines but underperformed structured clinical guidelines (Table 1). In Maiti et al., LLM-based tools scored below the EAN guideline on all items, with significant differences on two of three questions (Friedman *p* < 0.002) [18]. In Xiao et al., ChatGPT-4o in English scored highest and Kimi lowest across all criteria (all *p* < 0.001) [29]. Neither study quantified hallucination rates.

#### Patient and caregiver education

Eight studies addressed patient and caregiver education using three evaluation approaches: validated quality instruments [27, 30]; clinician-rated Likert frameworks [17–19, 26, 29]; and a usability-focused instrument [31]. Factual accuracy was generally high: Aguirre et al. reported 93% with zero fabricated or harmful responses [17]. However, quality fell below established sources. Dosso et al. found ChatGPT scored zero on the QUEST Attribution domain, meaning outputs lacked verifiable references entirely [30]. All readability assessments across all studies exceeded the recommended sixth-grade level [27, 30].

Customisation improved usability without improving accuracy. A RAG-grounded system achieved higher usability than baseline ChatGPT (CUQ 83.8 vs. 72.5, *p* = 0.005 for informativeness) [31], and prompt engineering improved actionability and relevance (*p* < 0.05) but not accuracy, trust, or safety [19].

Cross-language and evaluator-role differences emerged in Xiao et al., which contributed to both treatment guidance and education [29]. English responses outperformed Chinese on comprehensibility and specificity (both *p* < 0.001). Healthcare professionals rated responses higher than care partners on comprehensibility and actionability (both *p* < 0.001), while care partners were marginally more generous on accuracy (*p* = 0.11).

#### Cross-domain observations

**Fig. 2:**
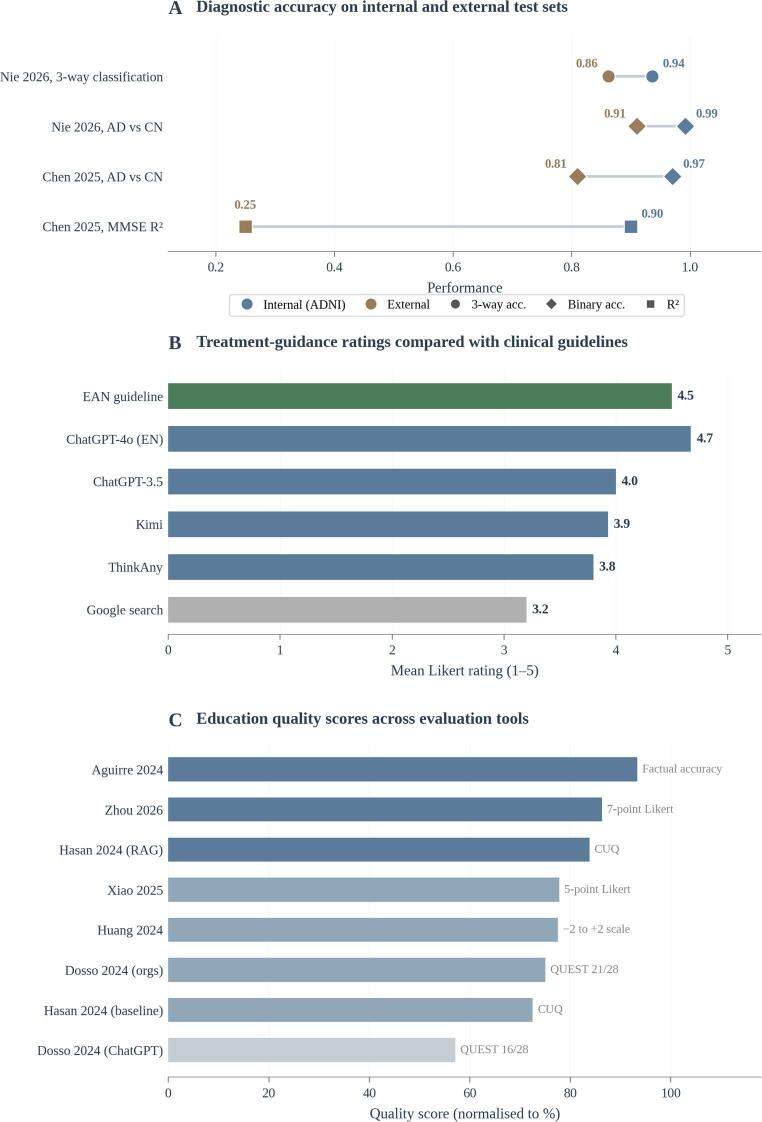
Summary of LLM performance across three clinical domains. (**A**) Diagnostic accuracy on internal (ADNI) versus external validation datasets. Nie 2026 reports three-way classification accuracy; Chen 2025 reports binary AD-versus-CN accuracy on OASIS (no MCI class). Zhang 2024 evaluated EHR extraction and is not plotted. (**B**) Mean Likert ratings for treatment-guidance quality compared with the EAN guideline (green). (**C**) Education quality scores normalised to a common percentage scale; evaluation tools shown beside each bar. Tukur Jido 2025 assessed readability only and is not included; Maiti 2025 is excluded from this panel because its single education question is not comparable to the multi-item instruments used by the other studies

Model performance was highest on internal validation datasets and declined on external data or clinically complex tasks. Safety data were sparse: only two studies quantified hallucination or error rates, and no study evaluated model outputs within clinical workflows. Figure 2 summarises LLM performance across all three domains (Table 2).

**Table 2:** Evidence map of LLM applications across clinical task domains in Alzheimer’s disease and mild cognitive impairment

Domain	Studies	Outcome instruments	Performance signal	Critical evidence gap
Diagnosis	3	Classification accuracy, AUC; R² for cognitive-score prediction; hallucination count (1 study)	High internal accuracy (0.94–0.97); 3-way classification dropped ~ 7 pp externally (Nie); binary classification dropped 16 pp on a different external task (Chen); cognitive-score R² collapsed 0.90 → 0.25	LLMs evaluated as classifiers, not as conversational diagnostic tools; no prospective use
Treatment guidance	2	Likert vs. structured guideline	LLMs below guideline (Likert 4.0-4.3 vs. 4.5), above search engines (3.1–3.7)	21 questions evaluated in total (3 from Maiti, 18 from Xiao); no biomarker-, stage-, or comorbidity-specific evaluation
Patient and caregiver education	8	DISCERN/QUEST, readability indices, CUQ, clinician-rated Likert	Moderate-to-high quality; weak source attribution; readability above 6th-grade threshold in every study reporting it	Heterogeneous instruments; no AD/MCI-specific attribution or readability adaptation; one RAG implementation

## Discussion

Across 11 studies, LLM outputs for AD and MCI received the highest ratings in patient and caregiver education and structured information extraction, and the lowest in diagnostic classification and treatment selection. No study evaluated prospective clinical use, and hallucination was quantified in only 2 of 11 studies. These findings are hypothesis-generating rather than practice-guiding.

### Safety and hallucination

The most important finding of this review concerns what remains unmeasured. Only two studies quantified hallucination or error rates. Zhang et al. reported 3 hallucinated MMSE values from GPT-4 versus 27 from LLaMA-2 [25], and Aguirre et al. reported zero fabricated responses across 60 caregiver queries [17]. The remaining nine studies did not systematically assess whether LLM outputs contained factual errors. This gap is the central obstacle to clinical application. In treatment guidance, a hallucinated drug interaction or fabricated contraindication could alter prescribing. In diagnosis, a confidently stated but incorrect cognitive score could shift staging. In patient education, unattributed or inaccurate information delivered to a cognitively impaired patient or an overwhelmed caregiver carries risks that differ from misinformation directed at a health-literate adult.

Evidence from outside this review reinforces these concerns. LLMs generate detailed responses to fabricated clinical inputs in up to 82% of cases [32], and differences in communication style alone can alter triage recommendations [33, 34]. In AD and MCI populations, where patients and caregivers vary in cognitive function, health literacy, and emotional state, sensitivity to prompt framing introduces a risk that none of the included studies evaluated.

### Task structure and domain-specific performance

Task structure shaped performance across all three domains. LLMs performed best where multiple acceptable outputs exist, such as drafting educational content or answering caregiver questions, and worst where a single verifiable answer is required.

In diagnosis, both vision-language models showed substantial internal-to-external performance drops, with MMSE-prediction R² falling to minimal predictive value externally [24, 28]. This pattern replicates established limitations of AI classifiers in dementia neuroimaging. Traditional CNNs show comparable internal-to-external drops despite architectures purpose-built for image classification [13]. That LLM-backbone models reproduce this failure mode confirms that the fundamental problem is dataset shift and population heterogeneity rather than model architecture [35, 36].

The clinical consequences extend beyond accuracy metrics. Distinguishing MCI due to AD from non-AD cognitive impairment (vascular, Lewy body, psychiatric) determines eligibility for disease-modifying therapies, prognostic counselling, and monitoring intensity. A model that achieves 0.86 three-way accuracy on a research dataset may perform differently when confronted with the heterogeneous presentations, comorbidities, and incomplete data typical of clinical practice. The evaluated systems function as classifiers, not conversational diagnostic tools. Conversational use, the modality through which LLMs are most likely to reach clinicians, remains unevaluated in AD or MCI.

Treatment guidance from LLMs consistently fell below structured clinical guidelines, with the gap widening as question complexity increased [18, 29]. This pattern has been observed across medical specialties [12, 14], but the clinical stakes in AD and MCI are disease-specific. Anti-amyloid therapies such as lecanemab and donanemab require biomarker-confirmed amyloid pathology, APOE ε4 genotyping for ARIA risk stratification, and serial MRI surveillance. Any incorrect intermediate output in this decision chain could alter eligibility or delay safety monitoring [7, 8]. Cholinesterase inhibitor prescribing involves cardiac comorbidity screening and interaction checks that depend on the patient’s full medication list. Management of behavioural and psychological symptoms of dementia requires integration of staging, comorbidity, and caregiver capacity that current LLMs cannot access. None of the included studies evaluated tasks requiring this level of patient-specific integration, and only 21 treatment questions were assessed across two studies.

Educational applications received the most consistent ratings but revealed two limitations specific to AD and MCI. LLM outputs lacked source attribution in every study, meaning caregivers receive guidance they cannot verify. This is a particular concern when the audience includes cognitively impaired patients and older adults with lower health literacy [37]. Readability exceeded recommended thresholds in every study that measured it [27, 30], a problem shared with existing health information [37] but particularly consequential in populations where comprehension affects medication adherence and safety decisions. LLM-generated educational content for these populations requires clinician review and readability adaptation before distribution.

### Customisation and retrieval-augmented systems

Prompt engineering and retrieval augmentation improved usability and engagement but did not improve clinical accuracy in either study that tested them [19, 31]. Both interventions changed how information was presented, not whether it was correct.

All included studies evaluate standalone or minimally customised LLMs without integration of external clinical data, real-time guidelines, or structured reasoning frameworks. The deficits observed across domains, including reduced external validity, lower guideline concordance, absent source attribution, and inability to incorporate patient-specific data, are problems that tool-augmented systems are designed to address. Evidence from other clinical fields suggests that retrieval augmentation can improve guideline grounding and source attribution [38, 39]. However, retrieval augmentation does not by itself ensure clinical correctness, appropriate reasoning, or safe patient-specific recommendations. The single RAG implementation in this review improved usability without improving accuracy, consistent with the possibility that augmentation enhances presentation more than clinical reliability [31]. In AD and MCI, where diagnostic criteria and treatment recommendations continue to evolve, knowledge-grounded systems may address some of these deficits, but clinician oversight remains necessary regardless of augmentation strategy [32].

### Strengths and limitations

This review evaluates LLMs across diagnosis, treatment guidance, and patient education in AD and MCI within a single synthesis. Screening used dual independent reviewers with blinded adjudication, and risk of bias was assessed with domain-appropriate tools (QUADAS-AI for diagnostic studies, AXIS for all others). Narrative synthesis followed the SWiM reporting guideline.

This review has several limitations. Although a larger evidence base was anticipated, only 11 studies met inclusion criteria. This reflects the limited number of studies evaluating LLMs in AD and MCI using clinically relevant outcomes. Many excluded studies focused on non-generative AI methods or reported technical outcomes without clinical relevance.

Heterogeneity is substantial, with at least 10 distinct evaluation frameworks and no standardisation among clinician-rated instruments. This prevented meta-analysis and limits cross-study comparability. Formal GRADE assessment was not applied because heterogeneity in outcome measures and absence of comparable effect sizes made certainty-of-evidence grading impracticable [14].

Most studies evaluate earlier-generation models, use synthetic prompts, and rely on convenience samples rather than real clinical interactions. These results characterise specific model versions, configurations, and prompting strategies evaluated at fixed points between 2024 and 2026, not LLMs as a category. Model capabilities are advancing in reasoning, multimodal processing, context length, retrieval integration, and source attribution, while AD and MCI clinical standards are themselves shifting, particularly around biomarker confirmation, anti-amyloid eligibility, APOE ε4 genotyping, and ARIA monitoring [4, 7, 8]. The evidence reviewed here should therefore be read as evaluations of particular model and clinical-knowledge environments, and any conclusion about a specific system will need to be revisited as both evolve. Three studies include general dementia cohorts under predefined inclusion criteria. Geographic representation is limited, with no studies from sub-Saharan Africa, South America, or South-East Asia. Cross-language conclusions are based on a single study. Publication bias toward positive findings is likely.

## Conclusions

Current evidence does not support the use of LLMs for diagnosis, treatment selection, or patient education in AD or MCI without clinician oversight; this conclusion reflects the systems and evaluation conditions reviewed rather than LLMs as a class. Before LLMs can be considered for routine clinical use, future studies should quantify hallucination rates systematically across application domains, evaluate diagnostic and treatment tasks against disease-specific decision chains including biomarker interpretation and anti-amyloid eligibility, and prospectively assess whether LLM outputs affect clinician decisions and patient outcomes within clinical workflows. As both model capabilities and clinical standards in AD and MCI continue to change, newer systems, particularly those incorporating retrieval augmentation, guideline grounding, source attribution, multimodal inputs, and patient-specific context, will require continued reassessment using standardised, disease-specific benchmarks and clinically realistic scenarios.

## Supplementary Information

Below is the link to the electronic supplementary material.

Supplementary Material 1
