# Supplementary material for: Generative large language models in the clinical management of Alzheimer’s disease and mild cognitive impairment
Source: Neurol Sci. 2026 Jul 13;47(8):626. doi: 10.1007/s10072-026-09239-2 (PMC13364785; doi:10.1007/s10072-026-09239-2)
Supplement: Supplementary file 1 — Supplementary Material 1 [file 10072_2026_9239_MOESM1_ESM.docx]

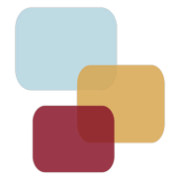


**BRIDGE GenAI Lab**

BIDMC–DFCI Radiology & Imaging Generative AI Hub

Beth Israel Deaconess Medical Center · Harvard Medical School

|  |  |  |
| --- | --- | --- |

# Supplementary Information

Generative Large Language Models in the Clinical Management of Alzheimer’s Disease and Mild Cognitive Impairment.

Contents

[Supplementary Information 1](#_Toc233471702)

[Supplementary Figures 3](#_Toc233471703)

[**Figure S1.** Risk-of-bias summary for included studies. (A) QUADAS-AI assessment of diagnostic studies (n = 3). (B) AXIS assessment of non-diagnostic studies (n = 8). 3](#_Toc233471704)

[Supplementary Tables 4](#_Toc233471705)

[**Table S1.** Search strategies across databases. 4](#_Toc233471706)

[**Table S2**. Risk-of-bias summary for all included studies (n = 11), including assessment tool, overall judgment, and primary methodological concern. 6](#_Toc233471707)

[**Table S3.** QUADAS-AI signalling questions and risk-of-bias judgments for diagnostic studies (n = 3). 7](#_Toc233471708)

[**Table S4.** QUADAS-AI justification notes for diagnostic studies (n = 3). 8](#_Toc233471709)

[**Table S5.** AXIS checklist responses for non-diagnostic studies (n = 8). 11](#_Toc233471710)

[**Table S6.** AXIS justification notes for non-diagnostic studies (n = 8). 13](#_Toc233471711)

[**Table S7.** Temporal and technical context of each included evaluation. 14](#_Toc233471712)

## Supplementary Figures

### **Figure S1.** Risk-of-bias summary for included studies. (A) QUADAS-AI assessment of diagnostic studies (n = 3). (B) AXIS assessment of non-diagnostic studies (n = 8).


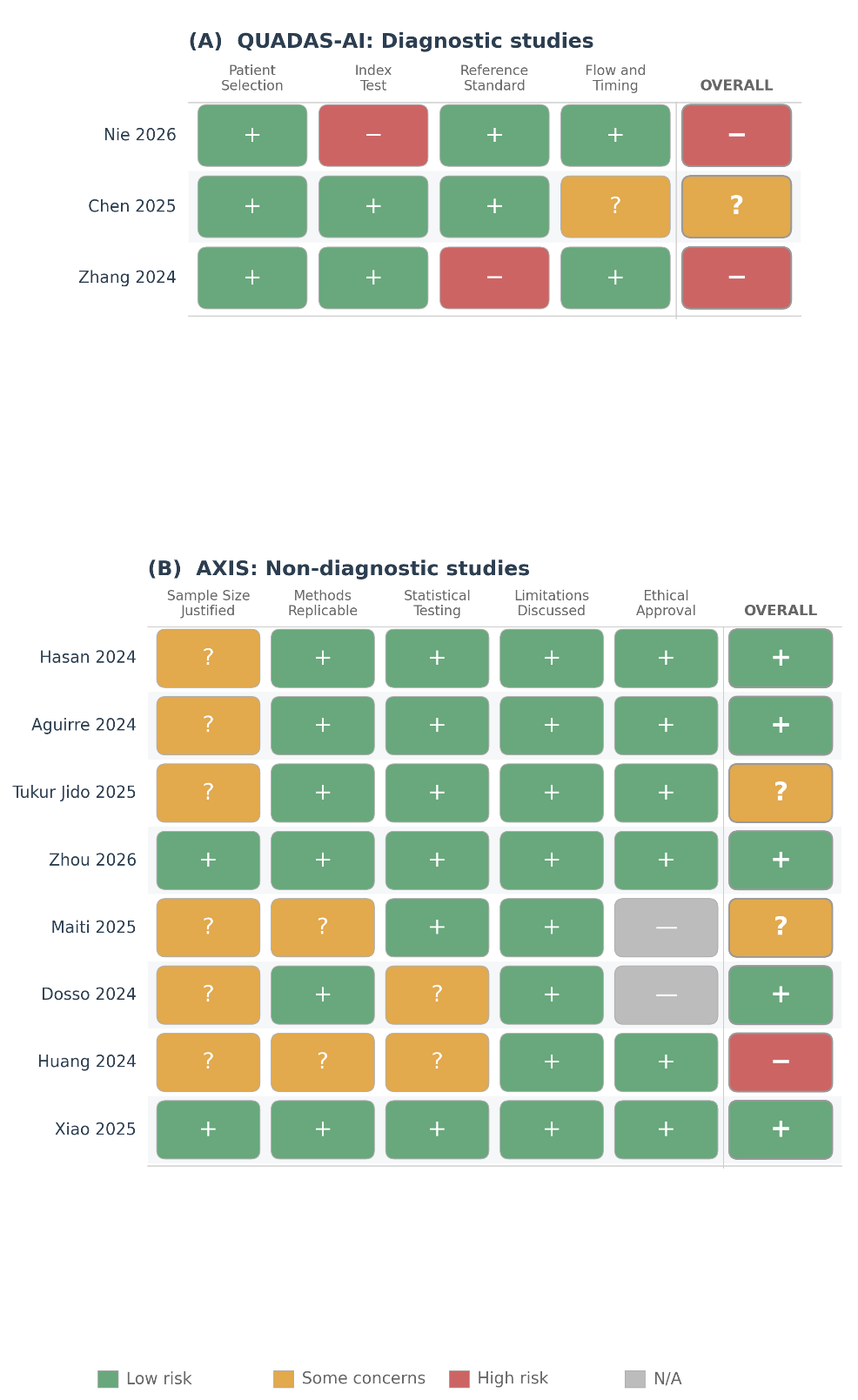

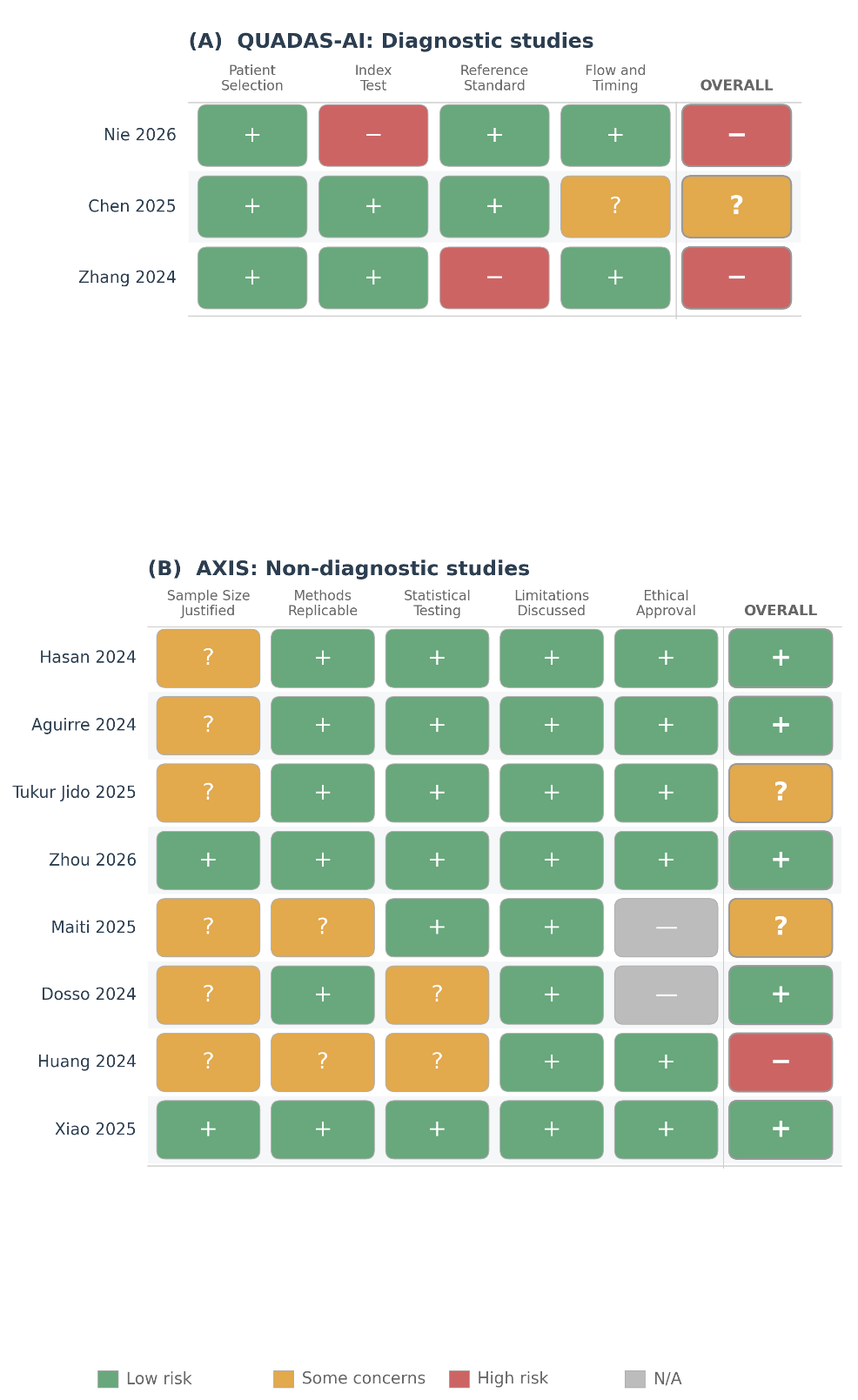

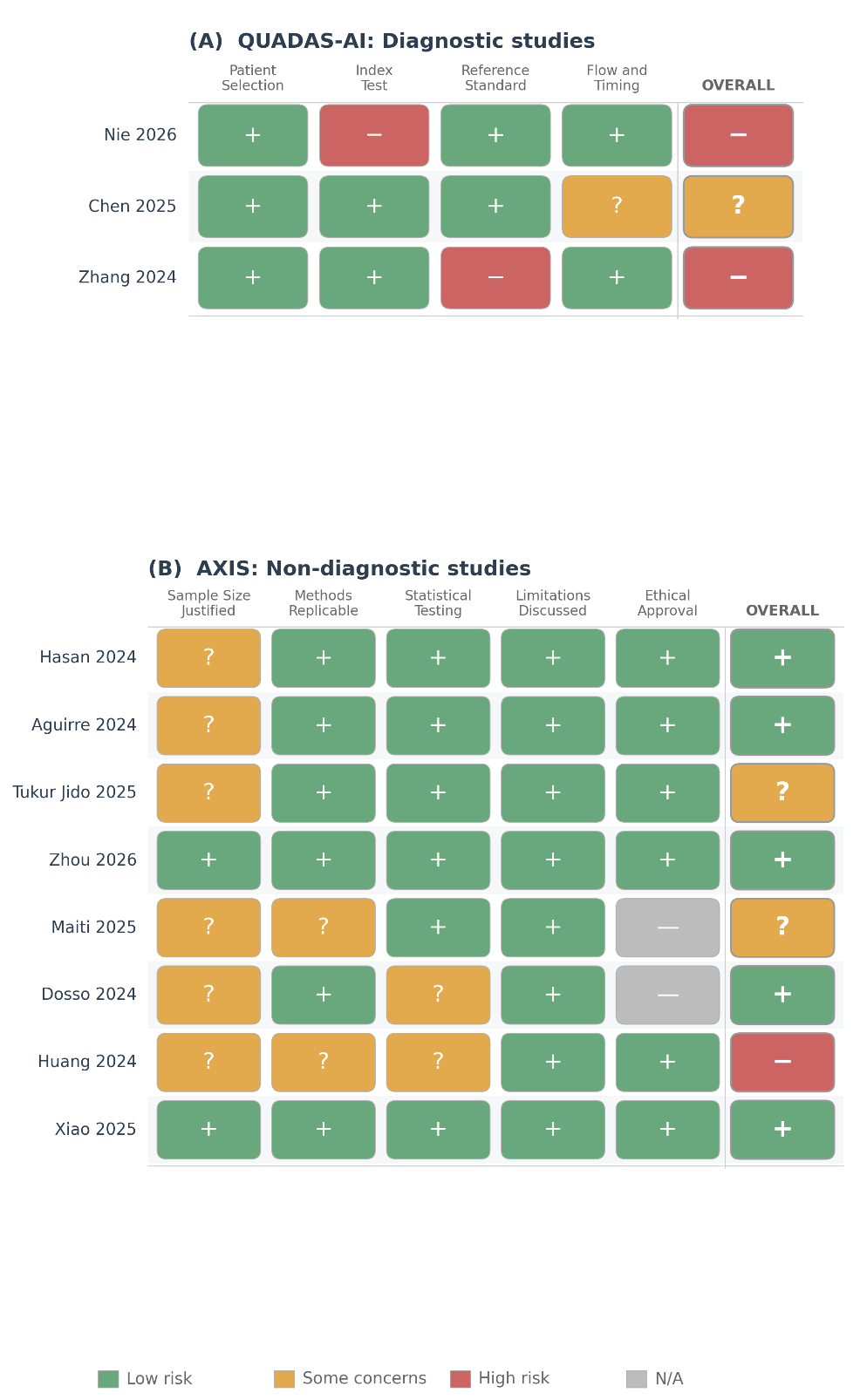


*Green circles indicate low risk of bias, yellow circles indicate some concerns, and red circles indicate high risk of bias.*

## Supplementary Tables

### **Table S1.** Search strategies across databases.

**PubMed**

(

"large language model"[Title/Abstract] OR "large language models"[Title/Abstract] OR LLM[Title/Abstract] OR LLMs[Title/Abstract] OR "generative AI"[Title/Abstract] OR "generative artificial intelligence"[Title/Abstract] OR "ChatGPT"[Title/Abstract] OR "GPT"[Title/Abstract] OR "GPT-4"[Title/Abstract] OR "GPT-3.5"[Title/Abstract] OR "GPT-4o"[Title/Abstract] OR "LLaMA"[Title/Abstract] OR "DeepSeek"[Title/Abstract]

)

AND

(

"Alzheimer"[Title/Abstract] OR "Alzheimer's disease"[Title/Abstract] OR "Alzheimer disease"[Title/Abstract] OR "mild cognitive impairment"[Title/Abstract] OR "MCI"[Title/Abstract] OR "cognitive decline"[Title/Abstract] OR "cognitive dysfunction"[Title/Abstract] OR "dementia"[Title/Abstract] OR "neurocognitive disorder"[Title/Abstract] OR "Alzheimer Disease"[MeSH] OR "Cognitive Dysfunction"[MeSH]

)

Filters: Language- English; Date: 2023/01/01 – Present

**PubMed Total: 279**

**Scopus**

TITLE-ABS-KEY

( (

"large language model" OR "large language models" OR "LLM" OR "ChatGPT" OR "GPT-4" OR "GPT-3.5" OR "GPT-4o" OR "generative artificial intelligence" OR "generative AI" OR "LLaMA" OR "DeepSeek"

)

AND

(

"Alzheimer" OR "Alzheimer's disease" OR "Alzheimer disease" OR "mild cognitive impairment" OR "MCI" OR "cognitive decline" OR "cognitive dysfunction" OR "dementia" OR "neurocognitive disorder"

) )

Filters: Language- English; Date: 2023/01/01 – Present

**Scopus Total: 587**

**PubMed Central**

(

"large language model"[Title/Abstract] OR "large language models"[Title/Abstract] OR "ChatGPT"[Title/Abstract] OR "GPT-4"[Title/Abstract] OR "GPT-3.5"[Title/Abstract] OR "GPT-4o"[Title/Abstract] OR "generative pre-trained transformer"[Title/Abstract] OR "LLaMA"[Title/Abstract] OR "DeepSeek"[Title/Abstract] OR "generative artificial intelligence"[Title/Abstract] OR "generative AI"[Title/Abstract]

)

AND

(

"Alzheimer Disease"[MeSH] OR "Cognitive Dysfunction"[MeSH] OR "Alzheimer"[Title/Abstract] OR "mild cognitive impairment"[Title/Abstract] OR "dementia"[Title/Abstract] OR "cognitive decline"[Title/Abstract] OR "neurocognitive disorder"[Title/Abstract]

)

Filters: Date: 2023/01/01 – Present

**PubMed Central Total: 231**

**Summary**

PubMed: 279 results

Scopus: 587 results

PubMed Central: 231 results

Total records before duplicate removal: 1097

Searches were conducted on 18 April 2026. Results may differ if the queries are re-run on a different date due to ongoing PubMed indexing updates.

### **Table S2**. Risk-of-bias summary for all included studies (n = 11), including assessment tool, overall judgment, and primary methodological concern.

| **Study** | **Tool** | **Overall RoB** | **Primary Concern** |
| --- | --- | --- | --- |
| Hasan 2024 | AXIS | Low | Small sample (n=20) but well-designed usability study with validated CUQ instrument and IRB approval. |
| Aguirre 2024 | AXIS | Low | 60 posts, 3 expert raters (>15 yr experience), consensus scoring, IRB approved. |
| Tukur Jido 2025 | AXIS | Some concerns | Very small item pool (n=3 per condition for AD subset). Automated readability only, no expert evaluation. |
| Zhou 2026 | AXIS | Low | Mixed methods, 12 experts, validated instruments, prompt engineering documented, IRB approved. |
| Maiti 2025 | AXIS | Some concerns | Only 3 questions. Seven evaluators (domain experts in dementia research). Methods insufficient for replication. Workshop paper. |
| Nie 2026 | QUADAS-AI | High | Best checkpoint selected on ADNI test set → test-set leakage. ADNI not representative of clinical populations. |
| Dosso 2024 | AXIS | Low | 18 FAQ items, QUEST validated tool, 2 coders with 83% inter-rater agreement resolved by discussion. |
| Chen 2025 | QUADAS-AI | Some concerns | Multiple visits of same patient treated as separate images; patient-level split stated but potential leakage ambiguous. |
| Zhang 2024 | QUADAS-AI | High | Ground truth = human-corrected ChatGPT output → reference standard contamination. Authors acknowledge. |
| Huang 2024 | AXIS | High | 6 of 10 evaluators are co-authors. Methods lack detail for replication. Co-author bias not discussed as limitation. |
| Xiao 2025 | AXIS | Low | 72 questions, 10 evaluators (6 HP + 4 CP), ICC for inter-rater reliability, IRB approved. |

### **Table S3.** QUADAS-AI signalling questions and risk-of-bias judgments for diagnostic studies (n = 3).

*Sounderajah V et al. Nat Med 2021;27:1663–5*

| **Signalling Question** | **Nie 2026** | **Chen 2025** | **Zhang 2024** |
| --- | --- | --- | --- |
| **1. Patient Selection** |  |  |  |
| Consecutive or random sample enrolled? | Low | Low | Low |
| Case-control design avoided? | Low | Low | Low |
| Inappropriate exclusions avoided? | Low | Low | Some concerns |
| AI: Dataset representative of target population? | Some concerns | Some concerns | Low |
| **→ RoB: Patient Selection** | Low | Low | Low |
| **→ Applicability: Patient Selection** | Some concerns | Some concerns | Low |
| **2. Index Test (LLM)** |  |  |  |
| Interpreted without knowledge of reference standard? | High | Low | Low |
| AI: Model version and access date reported? | Low | Low | Low |
| AI: Prompt design or fine-tuning reported? | Low | Low | Low |
| AI: Reproducibility addressed? | Low | Low | Some concerns |
| **→ RoB: Index Test** | High | Low | Low |
| **→ Applicability: Index Test** | Low | Low | Low |
| **3. Reference Standard** |  |  |  |
| Likely to correctly classify the condition? | Low | Low | Some concerns |
| Interpreted without knowledge of index test? | Low | Low | High |
| **→ RoB: Reference Standard** | Low | Low | High |
| **→ Applicability: Reference Standard** | Low | Low | Some concerns |
| **4. Flow and Timing** |  |  |  |
| Appropriate interval between tests? | Low | Low | Low |
| All patients received same reference standard? | Low | Low | Low |
| All patients included in analysis? | Low | Some concerns | Low |
| **→ RoB: Flow and Timing** | Low | Some concerns | Low |
| **OVERALL** |  |  |  |
| **→ OVERALL RISK OF BIAS** | High | Some concerns | High |

### **Table S4.** QUADAS-AI justification notes for diagnostic studies (n = 3).

| **Signalling Question** | **Nie 2026** | **Chen 2025** | **Zhang 2024** |
| --- | --- | --- | --- |
| **Patient Selection** |  |  |  |
| Consecutive/random sample? | ADNI: all eligible subjects with MRI+PET+clinical data. OASIS/NACC: entire available cohorts for external test. | Random stratified sample of 300 per class from ADNI by patient level. | Random selection of 765 from 34,465 eligible clinical notes. |
| Case-control avoided? | Registry-based cohort (AD/MCI/CN from ADNI), not case-control. | Same, ADNI registry, not case-control. | Cross-sectional EHR extraction. Not case-control. |
| Inappropriate exclusions? | No exclusions beyond data completeness requirements. | Subjects with missing MRI excluded; otherwise, appropriate. | Required brain MRI → excluded ~50% of patients. Racial composition shifts. Authors acknowledge. |
| AI: Representative dataset? | ADNI is predominantly white, highly selected research cohort. Not representative of real-world clinical populations. | Same ADNI limitation. OASIS zero-shot provides some external signal but also research cohort. | Real-world EHR from NYU Langone. Diverse academic medical centre. Racial breakdown reported. |
| **Index Test** |  |  |  |
| Blinded to reference standard? | Trained on labels; at inference no labels seen. BUT best checkpoint selected on ADNI test set → test-set leakage. | At inference, predictions generated without seeing labels. Patient-level split for test set. | Zero-shot prompting. No access to reference standard at any stage. |
| Model version reported? | LLaVA-1.5-7B, CLIP ViT-L/336px, Vicuna-v1.5. GitHub code link provided. | FLAN-T5, EVA_CLIP, bio-ClinicalBERT, ADFormer. GitHub code link provided. | GPT-4 API "2023-03-15-preview". LLaMA-2-70b-chat. Access date: June 9, 2023. |
| Prompt/fine-tuning reported? | LoRA fine-tuning. Prompt templates in Appendix A. All hyperparameters reported. | ADFormer fusion module. lr=2e-5, batch=8, AdamW, WarmupCosine scheduler. | Exact prompt provided in S3 Table. Temperature=0. All API parameters reported. |
| Reproducibility? | Code publicly available. Single training run; no variance across runs reported. | Code publicly available. Single run; no variance reported. | Single API session. Temperature=0 for determinism. No test-retest reliability. |
| **Reference Standard** |  |  |  |
| Correctly classifies condition? | ADNI clinical diagnoses per NIA-AA criteria. Well-validated. | ADNI clinical diagnoses + MMSE scores. Established instruments. | MMSE/CDR scores in notes are validated, but extraction ground truth derived from ChatGPT outputs. |
| Blinded to index test? | ADNI clinicians made diagnoses independently of AI model. | ADNI diagnoses made independently of AI model. | HIGH RISK: Reviewers saw ChatGPT outputs first, then corrected. Authors explicitly acknowledge bias toward GPT-4. |
| **Flow and Timing** |  |  |  |
| Appropriate interval? | Same ADNI visit timepoint for all data. | Same ADNI visit timepoint. | Extraction from same clinical note - no timing issue. |
| Same reference standard? | All classified by same ADNI diagnostic protocol. | All classified by same ADNI protocol. | All notes evaluated by same human review protocol. |
| All included in analysis? | All subjects with complete multimodal data included. | Multiple visits of same patient treated as separate images → potential patient-level train/test leakage despite stated patient-level split. | 765 sampled; 23 failed API extraction (742 remaining); 20 used for tuning (722 assigned to reviewers); 12 excluded for JSON parsing errors (710 in final analysis). All exclusions documented in study flowchart. |

### **Table S5.** AXIS checklist responses for non-diagnostic studies (n = 8).

*Downes MJ et al. BMJ Open 2016;6:e011458*

| **AXIS Item** | **Hasan** | **Aguirre** | **Tukur Jido** | **Zhou** | **Maiti** | **Dosso** | **Huang** | **Xiao** |
| --- | --- | --- | --- | --- | --- | --- | --- | --- |
| **Introduction** |  |  |  |  |  |  |  |  |
| 1. Aims/objectives clearly stated? | Yes | Yes | Yes | Yes | Yes | Yes | Yes | Yes |
| **Methods** |  |  |  |  |  |  |  |  |
| 2. Study design appropriate? | Yes | Yes | Yes | Yes | Yes | Yes | Yes | Yes |
| 3. Sample size justified? | No | No | No | Yes | No | No | No | Yes |
| 4. Target population clearly defined? | Yes | Yes | Yes | Yes | Yes | Yes | Yes | Yes |
| 5. Sample frame appropriate? | Yes | Yes | Yes | Yes | No | Yes | Yes | Yes |
| 6. Selection process representative? | Yes | Yes | Yes | Yes | No | Yes | No | Yes |
| 7. Non-respondents addressed? | N/A | N/A | N/A | N/A | N/A | N/A | N/A | N/A |
| 8. Outcome variables appropriate? | Yes | Yes | Yes | Yes | Yes | Yes | Yes | Yes |
| 9. Outcome variables measured correctly? | Yes | Yes | Yes | Yes | Yes | Yes | Yes | Yes |
| 10. Statistical significance clear? | Yes | Yes | Yes | Yes | Yes | No | No | Yes |
| 11. Methods enable replication? | Yes | Yes | Yes | Yes | No | Yes | No | Yes |
| **Results** |  |  |  |  |  |  |  |  |
| 12. Basic data adequately described? | Yes | Yes | Yes | Yes | No | Yes | Yes | Yes |
| 13. Non-response bias concerns? | N/A | N/A | N/A | N/A | N/A | N/A | N/A | N/A |
| 14. Results internally consistent? | Yes | Yes | Yes | Yes | Yes | Yes | Yes | Yes |
| 15. All planned analyses presented? | Yes | Yes | Yes | Yes | Yes | Yes | Yes | Yes |
| **Discussion** |  |  |  |  |  |  |  |  |
| 16. Conclusions justified? | Yes | Yes | Yes | Yes | Yes | Yes | Yes | Yes |
| 17. Limitations discussed? | Yes | Yes | Yes | Yes | Yes | Yes | Yes | Yes |
| **Other** |  |  |  |  |  |  |  |  |
| 18. Funding/COI declared? | Yes | Yes | Yes | Yes | Yes | Yes | Yes | Yes |
| 19. Ethical approval obtained? | Yes | Yes | Yes | Yes | N/A | N/A | Yes | Yes |
| 20. Future research addressed? | Yes | Yes | Yes | Yes | Yes | Yes | Yes | Yes |
| **Overall** |  |  |  |  |  |  |  |  |
| **OVERALL RISK OF BIAS** | Low | Low | Some concerns | Low | Some concerns | Low | High | Low |

Yes = Low risk | No = Concern | DK = Don’t know | N/A = Not applicable

### **Table S6.** AXIS justification notes for non-diagnostic studies (n = 8).

| **Study** | **Justification** |
| --- | --- |
| **Hasan 2024: Overall: Low** | IRB approved (NDSU, IRB0005069). 20 caregivers recruited via AD Advocacy and Support. Validated CUQ instrument. Wilcoxon signed-rank tests for paired comparisons. Prompts, RAG architecture, and knowledge graph documented. |
| **Aguirre 2024: Overall: Low** | IRB approved. 60 Reddit posts coded by 3 clinicians (>15 yr experience). Consensus scoring. 5-category quality framework (factuality, interpretation, application, synthesis, comprehensiveness). Limitations discussed. |
| **Tukur Jido 2025: Overall: Some concerns** | No human participants (AI-generated text only). AD subset n=3 per LLM, too small for stable estimates. Automated readability metrics only (FRE, FKGL, etc.). No expert content evaluation. Kruskal-Wallis test on 9 total outputs. |
| **Zhou 2026: Overall: Low** | IRB approved (George Mason University). 12 content experts. 32 scenario pairs (baseline vs prompt-engineered). Validated 9-item literature-based evaluation framework. Mann-Whitney U tests. Prompt engineering strategy fully documented. |
| **Maiti 2025: Overall: Some concerns** | Workshop paper (CEUR-WS). Only 3 clinical questions. Seven domain experts (2 mid-career, 3 established, 2 entry-level researchers). EAN guidelines as reference. Friedman test for overall comparisons. Methods lack sufficient detail for independent replication. |
| **Dosso 2024: Overall: Low** | 18 FAQ items across 3 North American AD organisations. QUEST validated quality tool. 2 coders with 83% inter-rater agreement, disagreements resolved by discussion. FKGL readability. No inferential statistics (descriptive only). No IRB stated, no human participants involved. |
| **Huang 2024: Overall: High** | IRB approved (Vanderbilt). 10 geriatricians evaluated 16 AD myths via REDCap survey. 6 of 10 evaluators are co-authors, which introduces potential bias. Likert scale descriptive only, no inferential statistics. Prompts not provided. Co-author evaluator overlap not discussed as a limitation (though other limitations are acknowledged). |
| **Xiao 2025: Overall: Low** | IRB approved (Tsinghua University, THU01KS2025035). 72 questions (18 treatment + 54 education). 10 evaluators (6 HP + 4 CP; 3 HP + 2 CP per language). ICC for inter-rater reliability. Kruskal-Wallis, Mann-Whitney U, Friedman tests. Bilingual design (EN/Chinese). All prompts documented. |

### **Table S7.** Temporal and technical context of each included evaluation.

Including publication year, model testing date or period, model and version, system type, external tool use, access to current clinical guidelines, and the guideline environment at the time of evaluation.

| **Study** | **Publication year** | **Model testing date or period** | **Model(s) and version** | **System type** | **External tools** | **Access to current guidelines** | **Guideline environment at evaluation** |
| --- | --- | --- | --- | --- | --- | --- | --- |
| Nie 2026 [24] | 2026 | NR (offline training and testing) | LLaVA-1.5-7B; CLIP ViT-L/14-336px; Vicuna-v1.5 (LoRA fine-tuned) | Fine-tuned / custom (open weights) | None | N/A (diagnostic classification) | N/A; reference = ADNI clinical diagnoses |
| Chen 2025 [28] | 2025 | NR (offline training and testing) | FLAN-T5 + EVA-CLIP + BioClinicalBERT (FLIQA-AD; fine-tuned) | Fine-tuned / custom (open weights) | None | N/A (classification and MMSE prediction) | N/A; reference = ADNI diagnoses and MMSE |
| Zhang 2024 [25] | 2024 | 9 June 2023 | GPT-4 (API "2023-03-15-preview"); LLaMA-2-70B-chat | Proprietary API (GPT-4) and open-source (LLaMA-2); zero-shot, temp 0 | None | N/A (information extraction) | N/A; reference = human-corrected model output |
| Maiti 2025 [18] | 2025 | 25 Apr – 12 May 2024 | ChatGPT-3.5; ThinkAny (AI search engine) | Consumer-facing (ChatGPT-3.5); AI search engine (ThinkAny) | ChatGPT-3.5: none; ThinkAny: web search | ChatGPT-3.5: embedded training only; ThinkAny: web retrieval | EAN dementia guideline (benchmark) |
| Xiao 2025 [29] | 2025 | Models selected by availability, Nov 2024 | ChatGPT-4o; Gemini 2.0 Flash; Kimi 1.18.1 (Moonshot) | Consumer-facing (official interfaces) | None | Embedded training only | Established guidelines from China and the UK (benchmark) |
| Hasan 2024 [31] | 2024 | NR | ADQueryAid (GPT-4 backbone + RAG + knowledge graph); baseline ChatGPT-3.5 | Custom pipeline (GPT-4 + RAG/KG); baseline consumer (ChatGPT-3.5) | RAG; knowledge graph (ontology); internal file retrieval | Yes - curated knowledge base of peer-reviewed literature and caregiving guidelines | Curated ADRD literature and guidelines (specific set NR) |
| Aguirre 2024 [17] | 2024 | NR (ChatGPT-3.5 cited as accessed Sep 2023) | ChatGPT-3.5 | Consumer-facing | None | Embedded training only | N/A (clinician-rated quality; no single-guideline benchmark) |
| Tukur Jido 2025 [27] | 2025 | Mar – Apr 2025 | GPT-4; DeepSeek-V3; Gemini Flash 2.5 | Consumer-facing (official interfaces) | None | N/A (readability only) | N/A (readability indices; no content evaluation) |
| Zhou 2026 [19] | 2026 | Sep – Nov 2024 (model use); interviews Jan 2025 | ChatGPT-4o (C1 baseline; C2 prompt-engineered) | Consumer-facing (C1); custom prompt-engineered (C2) | Prompt engineering (knowledge-augmented prompt); no retrieval | C1: embedded training only; C2: curated knowledge supplied in prompt | Reference grounded in NIH, NIA, Retirement Research Foundation, Korean Medical Association |
| Dosso 2024 [30] | 2024 | 11 Apr 2023 | ChatGPT (free version; GPT-3.5) | Consumer-facing | None | Embedded training only | N/A (QUEST tool; compared with 3 AD organisations' FAQs) |
| Huang 2024 [26] | 2024 | Apr 2023 – Feb 2024 | ChatGPT (GPT-4) | Consumer-facing | None | Embedded training only | N/A (clinician consensus on 16 AD myths) |

*AD, Alzheimer's disease; ADNI, Alzheimer's Disease Neuroimaging Initiative; ADRD, Alzheimer's disease and related dementias; API, application programming interface; CLIP, Contrastive Language-Image Pre-training; EAN, European Academy of Neurology; KG, knowledge graph; MMSE, Mini-Mental State Examination; N/A, not applicable; NIA, National Institute on Aging; NIH, National Institutes of Health; NR, not reported; RAG, retrieval-augmented generation; UK, United Kingdom. Studies are ordered by primary application domain (diagnosis, then treatment guidance, then patient and caregiver education); Maiti 2025 and Xiao 2025 also contribute to patient and caregiver education. "NR" denotes a field not reported in the source publication; dates are taken from each study as reported and were not inferred.*
